# Does generalized hypo-oxygenation (hypoxia) allow endotoxin into the brain through the blood brain barrier, thus increasing the risk for Parkinson disease?

**DOI:** 10.3325/cmj.2016.57.406

**Published:** 2016-08

**Authors:** JH Lange, Ines Niehaus, Luca Cegolon

**Affiliations:** 1 Envirosafe Training and Consultants, Pittsburgh, PA, USA *jhlange1@hotmail.com*; 2 independent contributor, Rendsburg, Germany,; 3 University of Padua, Department of Medicine, Padua, Italy; 4 London School of Hygiene and Tropical Medicine, Faculty of Epidemiology and Population Health, London, UK

To the Editor:

Recent laboratory research reported that in vitro hypo-oxygenation (hypoxia) can result in permeability changes of the blood brain barrier (BBB) (1). Such changes provide an opportunity for molecules (substances) normally excluded by the BBB to enter the brain and possibly be trapped, especially after the integrity of the BBB is restored. Some reports have suggested that endotoxin (lipopolysaccharide – LPS) may play a significant role in the occurrence of Parkinson disease (PD) (2,3). Experimental studies have shown that endotoxin reaching the brain can result in the destruction of cellular components and contribute to the induction of PD related events (4,5). In particular, both intra-cerebral exposure to LPS and systemic inflammation triggered by LPS seem to stimulate the production of pro-inflammatory cytokines in the brain leading to micro-glial activation, exacerbation of inflammation, and neuronal apoptosis (6). The dopaminergic neurons in the substantia nigra are more sensitive to the LPS-induced neurodegeneration because of the high density of microglia in the brain (7). Systemically injected LPS in a dose as low as 5 mg/kg in mice can result in chronic inflammation, leading to progressive destruction of the dopaminergic neurons in the substantia nigra (8). We are suggesting that LPS could be an “idiopathic” cause of PD due to endotoxin entering the brain as a result of a damaged (ie, leaky) BBB (9).

It has been shown that endotoxin itself can damage the BBB resulting in changes of permeability (10). Furthermore, one hour after i. v. injection of 1 μg/kg of LPS in rabbits the LPS concentration was 7.4 ± 2.2 pg/mL in the cerebrospinal fluid rising to 26.1 ± 5.2 pg/mL after three hours post injection, resulting in increased LPS in the brain due to BBB disruption (11). LPS can bind to the plasma membrane of microglia resulting in the occurrence of LPS-loaded vesicles and accumulation within the Golgi apparatus (12).

In addition, “movement” of endotoxin from the gastrointestinal tract to the brain has been proposed and may be enhanced through combination of other substances (eg, polychlorinated biphenyls) (13). Patients suffering from PD often have a leaky gut blood barrier with release of endotoxin from *Escherichia coli* (a Gram-negative bacterium) in the gut to the blood, resulting in reduced levels of LPS binding protein (in the blood), suggesting the occurrence of increased cytokine levels (14). LPS from the gut may be an important source of this agent during leaky periods of the BBB.

Hao et al indicated that the density of occludin and trans-endothelial electrical resistance were both reduced during hypoxic events as compared to controls (1). An event of this nature could allow endotoxin from the blood system to enter and become trapped in the brain. Occludin levels are also strongly reduced by the simultaneous effect of polychlorinated biphenyls and LPS (12).

Hypoxia can occur in cases of sepsis with a high release of endotoxin in the blood, creating a greater risk for developing parkinsonism and dementia in septic survivors (15). The role of LPS in the pathogenesis of other neurodegenerative diseases has been suggested based on plasma levels of LPS for Alzheimer disease (61 ± 42 pg/mL) and sporadic amyotrophic lateral sclerosis (43 ± 18 pg/mL), which is notably elevated when compared to controls (21 ± 6 pg/mL) (16).

It should be noted that a recent epidemiological publication evaluating endotoxin exposure did not find a relationship of this agent with PD (17). Rather, this study supports endotoxin having a protective benefit in occurrence of PD. But in this study plasma levels of LPS were not measured in agricultural workers as compared to a study of workers exposed to a high concentration of endotoxin in the air who experienced elevated plasma levels (0.1 to 0.45 endotoxin units/mL) and inflammation characteristics in the blood (18).

Based on the information provided by Hao et al (1), we would like to suggest that changes in the BBB permeability due to hypoxia could be one mechanism allowing endotoxin into the brain and contribute to idiopathic PD. There may also be events where exposure to other substances (12,19) may act in concert with LPS, resulting in PD type events.
